# Response of Plant Height, Species Richness and Aboveground Biomass to Flooding Gradient along Vegetation Zones in Floodplain Wetlands, Northeast China

**DOI:** 10.1371/journal.pone.0153972

**Published:** 2016-04-20

**Authors:** Yanjing Lou, Yanwen Pan, Chuanyu Gao, Ming Jiang, Xianguo Lu, Y. Jun Xu

**Affiliations:** 1 Northeast Institute of Geography and Agroecology, Chinese Academy of Sciences, Changchun, P. R. China; 2 School of Renewable Natural Resources, Louisiana State University Agricultural Center, Baton Rouge, United States of America; Chinese Academy of Sciences, CHINA

## Abstract

Flooding regime changes resulting from natural and human activity have been projected to affect wetland plant community structures and functions. It is therefore important to conduct investigations across a range of flooding gradients to assess the impact of flooding depth on wetland vegetation. We conducted this study to identify the pattern of plant height, species richness and aboveground biomass variation along the flooding gradient in floodplain wetlands located in Northeast China. We found that the response of dominant species height to the flooding gradient depends on specific species, i.e., a quadratic response for *Carex lasiocarpa*, a negative correlation for *Calamagrostis angustifolia*, and no response for *Carex appendiculata*. Species richness showed an intermediate effect along the vegetation zone from marsh to wet meadow while aboveground biomass increased. When the communities were analysed separately, only the water table depth had significant impact on species richness for two *Carex* communities and no variable for *C*. *angustifolia* community, while height of dominant species influenced aboveground biomass. When the three above-mentioned communities were grouped together, variations in species richness were mainly determined by community type, water table depth and community mean height, while variations in aboveground biomass were driven by community type and the height of dominant species. These findings indicate that if habitat drying of these herbaceous wetlands in this region continues, then two *Carex* marshes would be replaced gradually by *C*. *angustifolia* wet meadow in the near future. This will lead to a reduction in biodiversity and an increase in productivity and carbon budget. Meanwhile, functional traits must be considered, and should be a focus of attention in future studies on the species diversity and ecosystem function in this region.

## Introduction

Flooding regime is the most important factor potentially affecting wetland plant communities [1]. Natural and human activity-induced global changes (more intense rainfall events, longer dry periods, and artificial drainage from wetlands to periphery farmland) will have significant effects on flooding regimes in wetland ecosystems. It is therefore important to understand the impact of flooding depth on wetland vegetation across a range of flooding gradients. Many studies have investigated vegetation characteristics (including vegetation composition and distribution, species richness, biomass, and physiological ecology of dominant species) along flooding gradients (or vegetation zonation) [2–5]; however, the analysis of the underlying response mechanisms mainly focused on abiotic stress and competition. Few studies have incorporated the role of functional traits in the aforementioned pattern, although it has been proved to be a valid tool in explaining species diversity patterns [6].

As an overall assessment of plant stature, plant height is a quantitative trait which has been adopted by virtually everyone doing comparative plant ecology (Westoby et al., 2002 and references therein). It is central to a species’s carbon gain strategy by competitive advantage through prior access to light [7], and also influence reproductive biology (dispersal in particular) by flowers and seeds [8]. So the response of plant height to flooding condition might reflect their growth and adaptive characteristics. To date, the relative studies have been focused mainly on controlled conditions [9], with few studies quantifying individual species responses to natural flooding in the field.

Species richness is a quantified expression of species composition, and reflects the structure of the ecosystem in a certain extent. The pattern of species richness along flooding gradients has been the focus of ecology. The general pattern of intermediate effect is widely accepted [10, 11]. However, some researchers also observed positive or negative relationships between species richness and flooding depth [3, 12–14]; therefore, additional research needs to be incorporated into the debate. With regard to the driving mechanisms, biomass was often considered to impact species richness through regulating competition [15, 16]. In parallel, McGill et al. [6] advocated that functional traits should constitute the cornerstone for a more quantitative approach to biodiversity, and several studies disclosed that functional traits can capture more variations of species richness than abiotic stress/disturbance and biomass [12,17]. However, more empirical evidence is still lacking on the local scale.

Aboveground biomass, as the foundation of the food chain, providing habitat and forage for wildlife, furnishing shade and organic matter inputs to soil [3], is one of the main ecosystem functions. The pattern that community aboveground biomass decreases as flooding depth increases has often been observed [3, 18, 19], however, its universality needs more validation. The impact of flooding depth on community aboveground biomass has been confirmed by several studies [19, 20]. Some studies also have demonstrated that the key functional traits of dominant species and community level are the drivers of community biomass [12, 17, 21]. However, due to the difference of species composition, dominant species, and other environmental conditions, the relative contribution of flooding depth and key functional traits on biomass variation along flooding gradients depends on the quantifying analysis of specific sites.

In riparian and depressional wetlands of the Sanjiang Plain, Northeast China, the distribution of vegetation zones frequently occurs along elevation gradients. Within a relatively short horizontal distance (50–300 m), one can find large variations in water table depth from emergent marsh to meadow marsh and wet meadow. It is therefore an excellent place for studying species and community response to flooding gradients and for testing various ecological and biogeographical hypotheses. Several experimental studies of ecophysiological studies of individual species have identified the impact of water regime on wetland vegetation [22–24], but the results have limited utility in predicting species and community responses in natural settings.

The purpose of this study was to determine the response of three common vegetation communities in Northeast China, to the flooding gradient in riparian and depressional wetlands. Specifically, the study aimed to: 1) characterize the pattern of water table depth, plant height, species richness, and aboveground biomass along vegetation zones; 2) evaluate the relationship between water table depth and plant species richness, plant height, and aboveground biomass; and 3) determine major factors affecting species richness and aboveground biomass variation along a flooding gradient.

## Materials and Methods

### 2.1 Study sites

This study was conducted in five depressional wetlands and five riparian wetlands on the Sanjiang Plain, Northeast China (Table 1). These wetlands were located within several national nature reserves and along second-order tributaries to the upper Heilongjiang River. Mean annual precipitation in the area is approximately 550 mm, with 80% falling between May and September. Hydrologic conditions for the wetlands are dominantly influenced by rainfall and spring snowmelt, which typically begins in March or April, with peak flows in May. These wetland sites have been historically little affected by large-scale human activities.

**Table 1:** Wetland types, geographical location and the administration authority of the studied sites in Northeast China.

Sampling site	Wetland type	Site location	The authority responsible for the sampling site
Downstream of the Wusuli River	Riparian wetland	47.6462°N, 134.6310°E	Sanjiang National Nature Reserve Bureau
Downstream of the Naoli River	Riparian wetland	47.2931°N, 133.7747°E	Naoli River National Nature Reserve Bureau
Downstream of the Bielahong River	Riparian wetland	47.6203°N, 134.3109°E	Sanjiang National Nature Reserve Bureau
Yalu river	Riparian wetland	47.8744°N, 133.5775°E	Sanjiang National Nature Reserve Bureau
Nongjiang river	Riparian wetland	47.8298°N, 133.6946°E	Honghe National Nature Reserve Bureau
Sanjiang Marsh Experimental Station 1	Depressional wetland	47.5652°N, 133.5060°E	Northeast Institute of Geography and Agroecology, CAS
Sanjiang Marsh Experimental Station 2	Depressional wetland	47.5699°N, 133.5808°E	Northeast Institute of Geography and Agroecology, CAS
Honghe National Nature Reserve site 1	Depressional wetland	47.7756°N, 133.6611°E	Honghe National Nature Reserve Bureau
Honghe National Nature Reserve site 2	Depressional wetland	47.7335°N, 133.6080°E	Honghe National Nature Reserve Bureau
Dongfanghong National Nature Reserve	Depressional wetland	46.3957°N, 133.6262°E	Dongfanghong National Nature Reserve Bureau

Each of the study sites had a distinct vegetation zonation with a dominant plant community, such as emergent marsh, tussock marsh, and meadow marsh. The vegetation communities occurred along a moisture gradient in elevation from the stream bank to the floodplain terrace. The emergent marsh vegetation (referred to in the following as the *Carex lasiocarpa* community) was typically along streamsides or in depressional centres and was periodically flooded each spring for several weeks because of low elevation. The wet meadow vegetation (the *Calamagrostis angustifolia* community) occupied higher floodplain terraces, which were flooded only occasionally. The meadow marsh vegetation (the *Carex appendiculata* community) grew in the floodplain terrace areas with intermediate elevation and, hence, was partially flooded during growing season. These three communities once covered more than 51% of the wetland area in this region [25]. Details on vegetation composition and soil properties along the zonation can be found in Lou et al. [26].

### 2.2 Vegetation survey

We conducted vegetation survey in August 2012 at five depressional wetlands and five riparian wetlands in the Sanjian Plain. At each of the depressional wetland sites, to cover the full water-level gradient, two transects connecting through the center of the depression were placed from the one side upper wet meadow to the other upper wet meadow randomly; similarly, at each of the riparian wetland sites, two transects were placed perpendicular to the river; therefore, a total of 20 transects were laid out. Along each transect, three 1 m by 1 m plots were identified for each of the three vegetation communities, making a total of 180 plots for the study (i.e., 60 plots for each community). The plot locations were subjectively chosen. Sampling was both purposive (to sample different marsh community) and opportunistic (locations were chosen based on ease of access). Presence of all rooted species, visual estimates of cover for each species, and water table depth were recorded for each plot. Plant height was measured for about 20 randomly-selected individuals of dominant species. The height measurements were used as the maximum height reached by the individuals during the growing season. The plant nomenclature follows by Fu [27].

In addition, plant above-ground biomass was determined from a 0.5 m by 0.5 m plot within each 1 m by 1 m plot along a transect. All litter and rooted vegetation within the plots were clipped to the ground surface, and the materials were collected, air-dried, sorted into each species, and then oven dried for 72 h at 65°C, and finally weighed.

### 2.3 Water level monitoring

In early June 2011, a transect was established for each of the three dominant herbaceous plant communities at one depressional wetland. Dip wells were placed in each plant community along the transect and piezometers were constructed using 8-cm diameter by 1.5-m long PVC pipes, which was drilled with 0.32-cm diameter holes along the entire buried length. Each piezometer was capped and placed along the transect in a hole bored with a standard soil auger to a depth of approximately 1 m. Water level was recorded every two hours during the growing seasons of 2011 and throughout 2012 with a pressure transducer data logger of 1 cm accuracy. The logger was placed hanging in the piezometer pipe.

During vegetation investigation, water table tubes were inserted adjacent to each quadrat, and water table position was measured on the second day after the investigation.

Flooding depth records were used for comparison of flooding depth of three communities. To note, the study area received an annual total precipitation of 324 mm in 2011 and of 456 mm in 2012, both of which were below the area’s long-term average precipitation.

### 2.4 Ethics statement

All our field work in each sampling site was given permission by the corresponding authorities responsible for the sampling sites (Table 1). No specific permits were required for our field studies, and the field study did not involve any endangered or protected species.

### 2.5 Data analysis

Water table variables were selected to quantify the observed patterns with a focus on habitat wetness during the growing season. Maximum, minimum, and mean water table, dynamic variable ranges were subsequently calculated for each community type. Before calculating these summary statistics, mean daily water levels were first summarized as the mean of recorded values of every two hours for each 24-hour period. One way ANOVA was used to test change in water level for each plot between the two surveys.

For each quadrat, a mean height value was calculated using the height value of each species weighted according to the species relative abundance in the community as follows:

$$\sum_{k\;=\;1}^{n_{j}}A_{k,j}\times T_{k,j}$$

Where nj is the number of species sampled in community j, Ak,j is the relative abundance of species k in community j, and Tk,j is the height of species k in community j.

A one-way ANOVA was used to test for community differences along vegetation zone in mean water table depth, species richness, and aboveground biomass. Multiple comparisons were tested using Turkey’s honest significant difference (HSD). Regression analyses were used to test the pattern of dominant species height (average stem height), species richness, and aboveground biomass along flooding gradient. When each community was analysed separately, stepwise regression was used to test the effect of water table depth, dominant species height, community mean height, and aboveground biomass on species richness, as well as the effect of the first three above-mentioned factors and species richness on aboveground biomass. When all three communities were combined, a general linear model (GLM) was used to quantify the effects of water table depth, dominant species height, community mean height, and aboveground biomass on species richness, and the effects of the first three aforementioned factors and species richness on aboveground biomass. Water table depth, dominant species height, community mean height, and aboveground biomass/species richness were treated as independent variables, and community type was treated as a covariant to account for the non-independence of species richness/aboveground biomass within a community. Given that dominant species height, community mean height, and aboveground biomass are highly correlated with each other, only one of them was included in each main-effect model to avoid multiple collinearity in GLM. Variables with significant effects on species richness/aboveground biomass and interaction terms of these variables were included in the final model. All the above-mentioned analyses were carried out using R 2.12.0 (R Development Core Team 2010).

## Results

### 3.1 Hydrologic conditions

**Fig 1:**
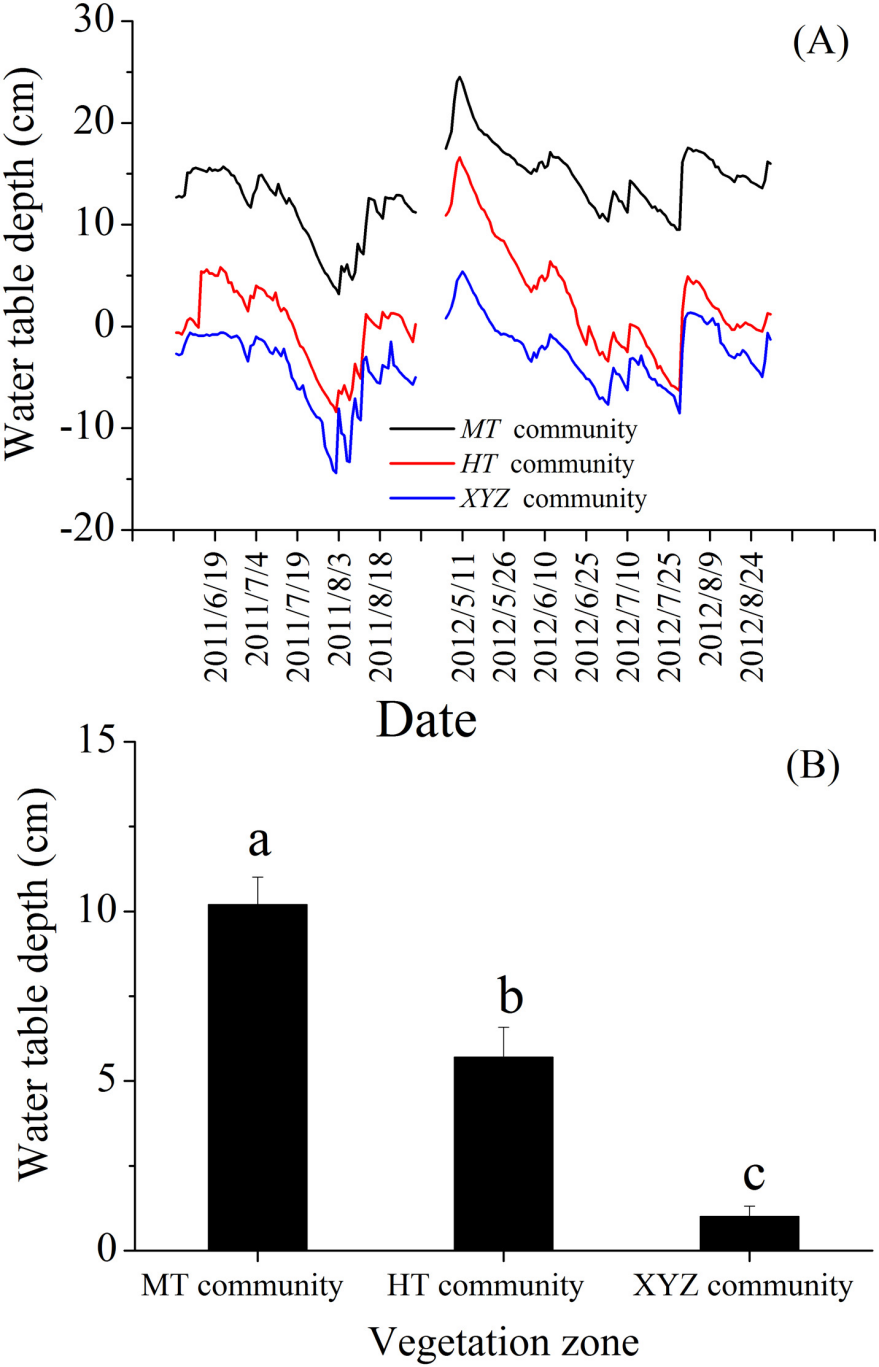
Water table depth patterns for *Carex lasiocarpa*, *Carex appendiculata*, and *Calamagrostis angustifolia* community along vegetation zone. A for growing season pattern in 2011 and 2012; B for mean water table depth measured during vegetation investigation. Error stick denotes one standard error (SE), a, b, c denotes Tukey result of post hoc comparison. MT, *Carex lasiocarpa*; HT, *Carex appendiculata*; XYZ, *Calamagrostis angustifolia*.

Spatially, flooding depth gradually decreased from the *C*. *lasiocarpa* community to the *C*. *angustifolia* community as observed in both the continuous water table monitoring (Fig 1(A)) and the on-site measurements during vegetation sampling (Fig 1(B)). The difference in water table depth among the three communities is statistically significant. During the 2-year study period, the water table was highest in May, the beginning of a growing season, due to spring snowmelt, followed by a 10- to 15-cm drop in the dry month of June. Then, water table depth increased by approximately 10 cm in response to the large rain events during July and August. Throughout the growing seasons of 2011 and 2012, the water table in the *C*. *lasiocarpa* community remained above the ground surface. However, during the same period, the water table in the *C*. *appendiculata* community and in the *C*. *angustifolia* community was above the ground surface for only one month and for a few days, respectively. There was a large fluctuation (approximately 20- to 25- cm) in the water table change within each of the three communities.

### 3.2 Height of dominant species

Heights of the dominant species ranged from 35 to 210 cm (Table 2). Significant differences in the height among three communities were found for *C*. *lasiocarpa* (ANOVA; F_2,101_ = 14.074, p < 0.001) and *C*. *angustifolia* (ANOVA; F_2,100_ = 13.884, p <0.001), but not for *C*. *appendiculata* (ANOVA; F_1,73_ = 3.244, p = 0.076).

**Table 2:** Height of dominant species and community mean of the three marsh communities.

Community	Species	Number of plots	Height of dominant species (cm)			Community mean height (cm) (mean ± SE)
			Min	Max	Mean (±SE)	
***Carex lasiocarpa***	*Carex lasiocarpa*	60	50	105	83.9 ± 1.4a	69.5 ± 1.36a
	*Calamagrostis angustifolia*	10	50	90	67.5 ± 4.0a	
	*Carex appendiculata*	60	48	115	70.3 ± 1.9	
***Carex appendiculata***	*Calamagrostis angustifolia*	33	40	110	80.7 ± 3.2b	67 ± 1.7a
	*Carex lasiocarpa*	24	50	100	73.0 ± 2.6b	
	*Calamagrostis angustifolia*	60	60	210	100.5 ± 3.3c	
***Calamagrostis angustifolia***	*Carex lasiocarpa*	20	35	104	66.9 ± 4.6c	89.1 ± 3.15b
	*Carex appendiculata*	15	40	90	62.7 ± 3.8	

a, b, c denotes Tukey result of post hoc comparison.

**Fig 2:**
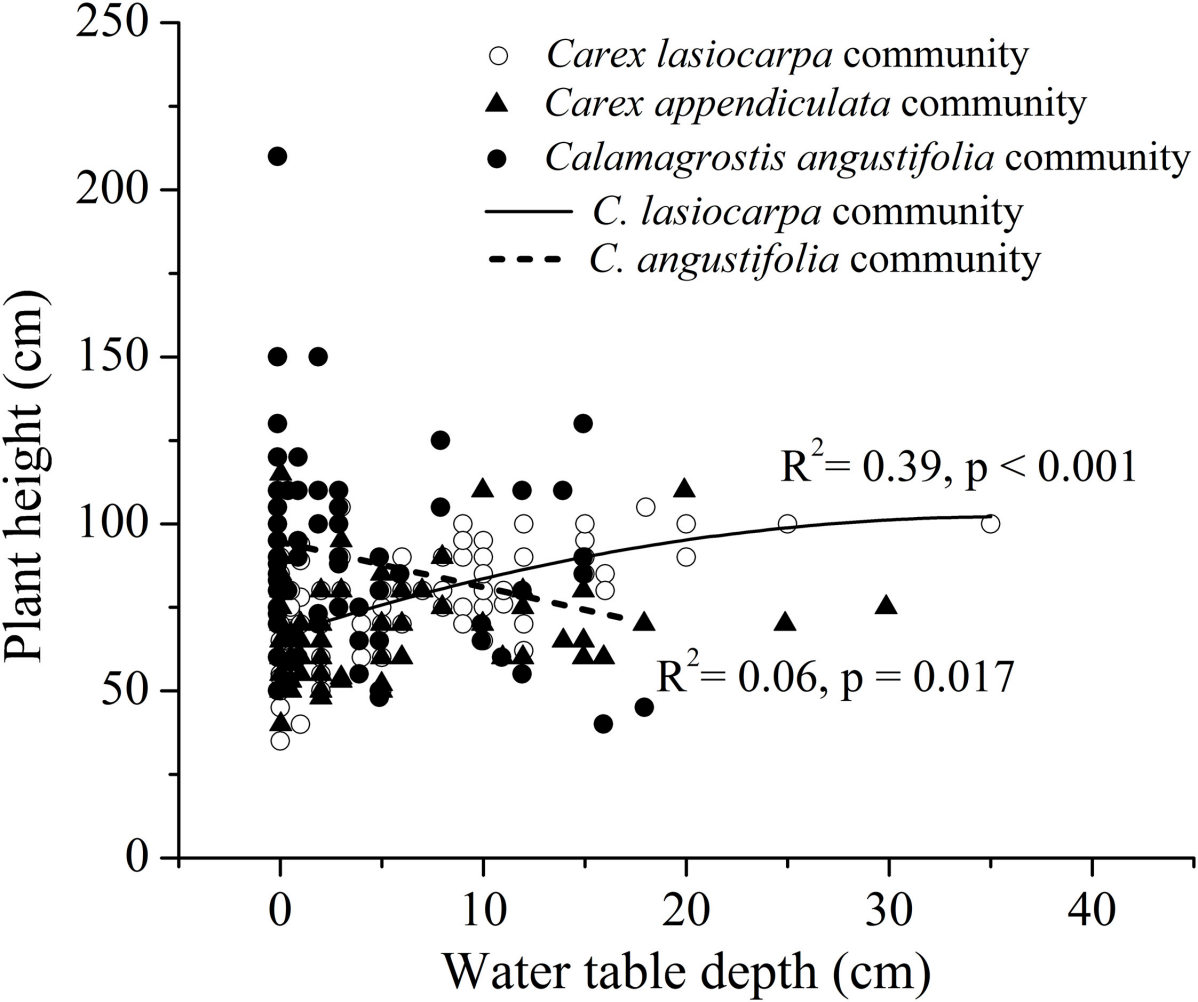
The relationship between average plant height and water table depth for three dominant species ((A) *Carex lasiocarpa* (B) *Carex appendiculata* (C) *Calamagrostis angustifolia*).

The height of *C*. *lasiocarpa* showed parabolic form, but basically showed an increasing trend with increasing water table depth in our data range (R^2^ = 0.39, F = 31.972, p < 0.001), while *C*. *angustifolia* displayed a negative correlation between height and water table depth (R^2^ = 0.05, F = 5.945, p < 0.02). No relationship between the two parameters was found for *C*.*appendiculata* (Fig 2).

### 3.3 Species richness

Species richness varied from 3 to 17 and differed significantly between the *C*. communities and the *C*. *angustifolia* communities (Table 3), with the highest value in the *C*. *appendiculata* community and the lowest value in the *C*. *angustifolia* community (Table 3).

**Table 3:** The describe statistic of species richness and biomass for three herb marsh communities.

Community	Species richness			ANOVA		Aboveground biomass (g/m^2^)			ANOVA	
	Min	Max	Mean (±SE)	F(2,178)	P	Min	Max	Mean (±SE)	F(2,178)	P
*Carex lasiocarpa*	3	11	7.77 ± 0.25a			194.33	699.23	385.30± 6.04a		
*Carex appendiculata*	4	17	8.58 ± 0.40a	13.615	< 0.001	210.15	835.93	487.23± 0.73b	40.934	< 0.001
*Calamangrostis angustifolia*	4	15	6.28 ± 0.27b			240.00	1289.13	692.11± 3.68c		

a, b, and c denote Tukey result of post hoc comparison.

**Fig 3:**
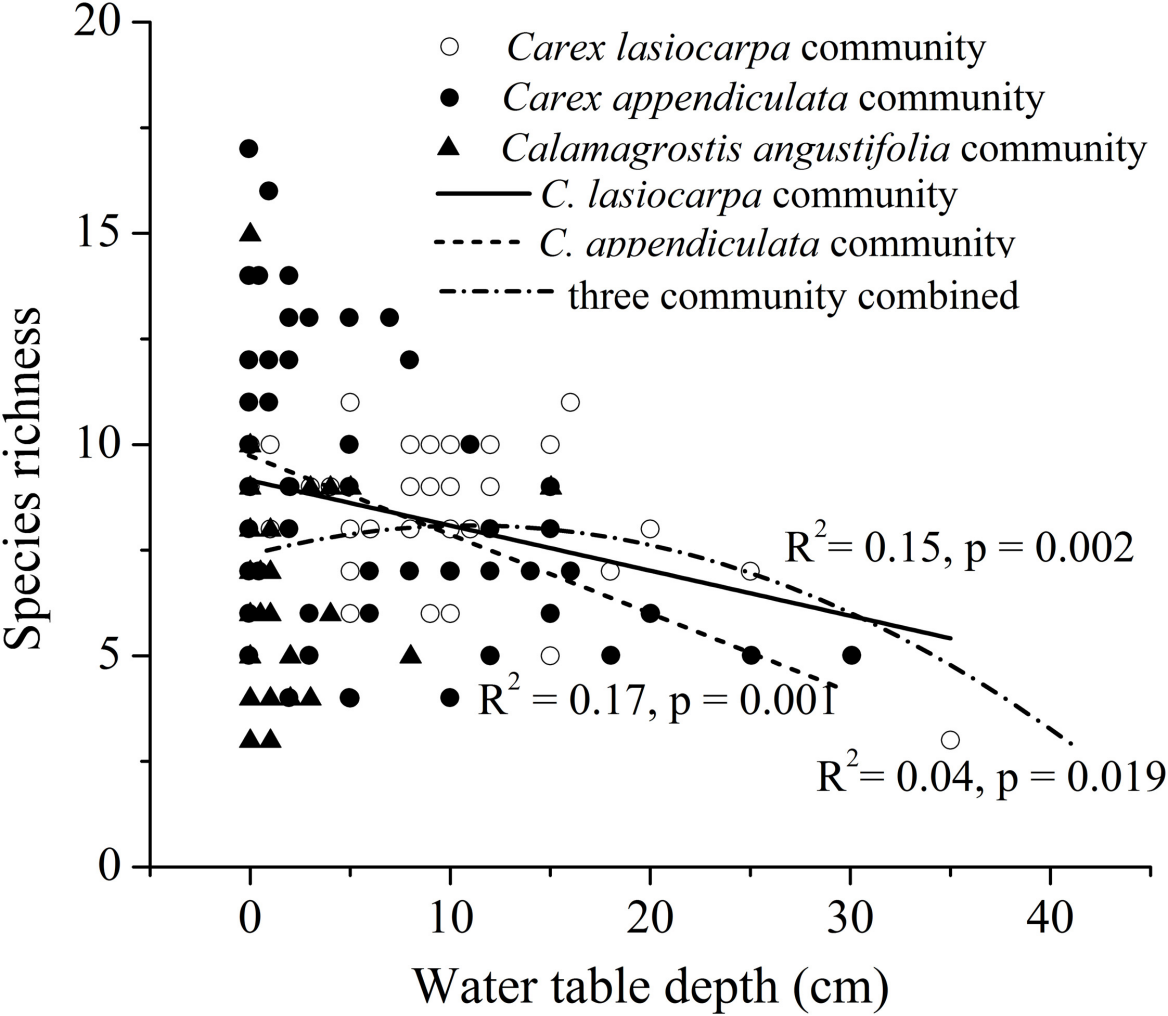
The relationship between species richness and water table depth. (A) *Carex lasiocarpa* community, (B) *Carex appendiculata* community, (C) *Calamagrostis angustifolia* community, (D) all three communities combined.

A clearly linear, negative correlation between water table depth and species richness was found for the *C*. *lasiocarpa* community (R^2^ = 0.14, F = 10.417, p = 0.002; Fig 3(A)), and the *C*. *appendiculata* community (R^2^ = 0.15, F = 11.710, p = 0.001; Fig 3(B)), but no significant correlation for the *C*. *angustifolia* community (Fig 3(C)). There was, however, a significant hump-shaped response for the three communities combined (R^2^ = 0.04, F = 4.042, p = 0.02; Fig 3(D)).

### 3.4 Aboveground biomass

Aboveground biomass in the 180 sampling plots varied by one order of magnitude, from 194 to 1489 g m^-2^, and differed significantly among the three communities (Table 3). The *C*. *angustifolia* community was the most productive, followed by the *C*. *appendiculata* community. The *C*. *lasiocarpa* community showed the lowest aboveground biomass (Table 3).

**Fig 4:**
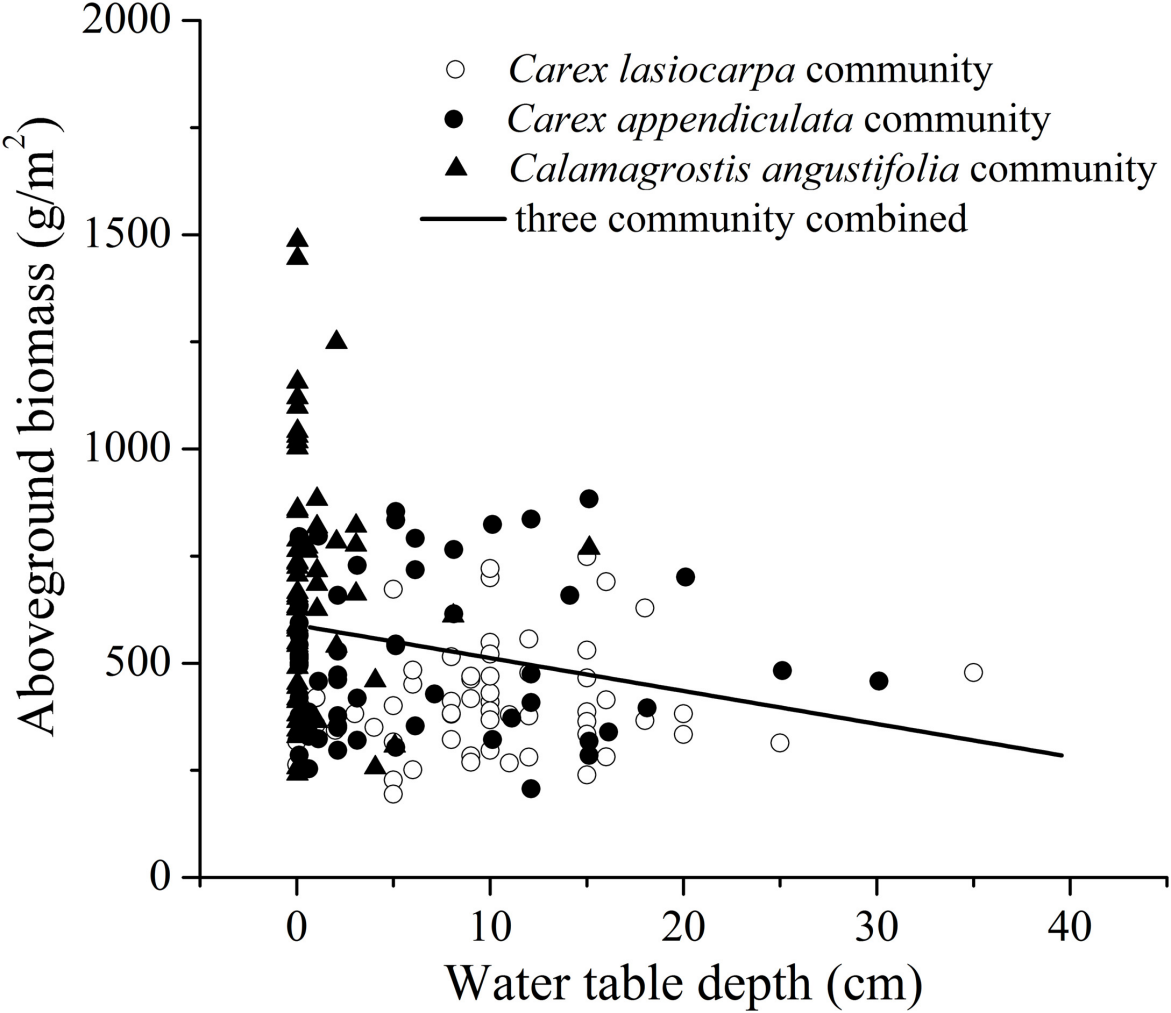
The relationship between aboveground biomass and water table depth. (A)–(C) are for *Carex lasiocarpa*, *Carex appendiculata*, and *Calamagrostis angustifolia* community, (D) is for all three communities combined.

No significant correlation between these two parameters was found for each community (Fig 4(A)–4(C)). However, a significantly negative correlation between water table depth and aboveground biomass was found for the three communities combined (R^2^ = 0.05, F = 11.001, p = 0.001; Fig 4(D)).

### 3.5 Analysis of the controlling factor of species richness and aboveground biomass

When communities were analysed separately, stepwise regression analysis revealed that only water table depth had a significant effect on species richness in two *C*. communities (Table 4), and only dominant species height had a significant effect on the aboveground biomass in the three communities (Table 4). When the three communities were combined, GLM analysis showed that for species richness, only community type, water table depth, and community mean height were included in the final model (Table 5), with these variables explaining 8.5%, 7.8%, and 5.2% of the variation in species richness, respectively. For abovupground biomass, the best model included dominant species height and community type (Table 5), which explain 23.8% and 29.4% of the variation in aboveground biomass, respectively (Table 5).

**Table 4:** Multivariate stepwise regression analysis for species richness and aboveground biomass.

Response variable	Community	Explanation variable	R^2^	F	P
	*Carex lasiocarpa*	**WD** + DSH + CMH + AB	0.14	10.417	0.002
**Species richness**	*Carex appendiculata*	**WD** + DSH + CMH + AB	0.15	11.710	0.001
	*Calamagrostis angustifolia*	WD + DSH + CMH + AB			
	*Carex lasiocarpa*	WD + **DSH** + CMH + SR	0.17	13.383	0.001
**Aboveground biomass**	*Carex appendiculata*	WD + **DSH** + CMH + SR	0.28	23.672	< 0.001
	*Calamagrostis angustifolia*	WD + **DSH** + CMH + SR	0.58	81.250	< 0.001

Explanation variables in bold were significant variables with p < 0.05. WD, water table depth; DSH, dominant species height; CMH, community mean height; AB, aboveground biomass; SR, species richness.

**Table 5:** Summary of general linear models for species richness and aboveground biomass.

Factor	Species richness				Aboveground biomass			
	Main-effect model			Final model	Main-effect model			Final model
	DF	MS	F	SS%	DF	MS	F	SS%
Covariance								
CT	1	104.533	**19.0895**	8.5%	1	2391063	**90.5437**	23.8%
Abiotic factor								
WD	1	94.988	**17.3482**	7.8%	1	16892	0.6397	
Biotic factor								
DSH	1	6.79	1.2391		1	2948435	**111.650**	29.4%
CMH	1	64.28	**11.7381**	5.2%	1	72924	2.7614	
AB	1	1.83	0.3349					
SR					1	8843	0.3349	

CT, community type; WD, water table depth; DSH, dominant species height; CMH, community mean height; AB, aboveground biomass; SR, species richness.

## Discussion

Significant differences in the hydrologic conditions found in this study provide a good flooding gradient among three plant communities along the sampled transects. Quantifying the response of plant height, species richness, and aboveground biomass to flooding gradient is important for ecologists to understand the driving mechanism of these patterns.

### 4.1 Responses of plant height to flooding gradient

The changing patterns of plant height of the three species along the water table depth gradient suggested that the plasticity in plant growth responses to hydrological regime were species-specific. Specifically, flooding significantly facilitated the growth of *C*. *lasiocarpa*. This demonstrates that *C*. *lasiocarpa* is a flood-tolerant species with relatively high porosity [28] that mainly establishes in permanently inundated habitats [29], yet high waterlogging stress would obviously restricted the growth of *C*. *lasiocarpa*, and this is in line with the conclusions of artificial simulation experiment carried out by Shi et al [30]. However, the height of *C*. *angustifolia* was noticeably inhibited by flooding stress. This observation is consistent with some studies in which this flood-sensitive species is identified as having a limited potential for oxygen diffusion, and thus it is primarily distributed in high elevation places [24, 29, 30]. The response trend of *C*. *appendiculata* requires further study based on more field observations and analyses because the lack of a significant correlation in our study may be related to an incomplete water table depth gradient and the relatively small sample size.

However, water table depth could only possibly explain at most 37% of the total variation in the data set (Fig 2(A), R^2^ = 0.37). This suggests that except water table depth, other habitat factors, e.g., light intensity, which has been confirmed to play an important role in determining plant growth and distributions in this region [22]. In addition, temperature could also be important, especially because the study sites were located in a cold region with an annual average temperature of 2.1°C.

### 4.2 Response of species richness to flooding gradient

Our studies indicated a unimodal relationship between species richness and water table depth (Fig 3), and this is consistent with the moderate hypothesis and the findings of several other local studies (e.g., [31, 32]). In fact, the unimodal relationship in our study resulted from the negative correlation of two *Carex* communities and no significant correlation of the *C*. *angustifolia* community on a smaller scale. Apparently, negative correlations in the two *Carex* communities may be related to flooding stress, e.g., at the very wet end of flooding gradient, species richness is limited as only few species are adapted to deep-flooding conditions. While no significant correlation occurred in the *C*. *angustifolia* community, this may have resulted from the fact that plant competition for light and space in occasionally flooded habitats (the dry end of flooding gradient) leads to a competitive exclusion of species; hence, the recruitment of species was not primarily controlled by water table depth, but may depend more on biotic interactions (inter-specific competition). This also confirms earlier work that species richness of communities at relatively lower elevations is controlled by abiotic stress in the flooded zone and by plant interactions at relatively higher elevations [4, 33–35].

When analyzing jointly other biotic factors (community type, dominant species height, community mean height, aboveground biomass), water table depth still has more stronger effect power on species richness (Table 4). However, it only explain about 15% variation of species richness. Several studies [15, 36, 37] reported that competition, species pool, and light intensity could play a role in controlling species coexistence in marsh vegetation. Therefore, further study is needed to elucidate the question.

In addition, the negative correlation between species richness and water depth in two *Carex* communities indicated that species number per plot would show increasing trends with the habitat drying, similar to the result of Dwire et al. [38], and confirmed by a long-term study by Lou et al [39]. Furthermore, this also demonstrated that the water table depth of the growing season is a significant predictor of species diversity for marsh vegetation.

### 4.3 Response of aboveground biomass to flooding gradient

For each community, aboveground biomass was not affected by water table depth (Fig 4). This contrast with the conclusions of artificial simulation experiment implemented by Zhang et al. [40] and Shi et al. [30], in which water level significantly affect aboveground biomass of dominant species *C*. *lasiocarpa* and *C*. *angustifolia*. This is related to the fact that aboveground biomass of a community is determined by species composition and stem density, except growth forms of dominant species [3]. When three communities are combined, the negative correlation between water table depth and aboveground biomass was shown. Significantly, this is a pseudo-correlation statistically, and resulted from the fact that aboveground biomass increased as flooding depth decreased along the vegetation zone. So with the habitat drying resulted from human activity and climate warming in this region marshes [39], vegetation productivity and carbon sequestration in this temperate freshwater marshes would increase by species replace and community succession.

From abiotic factors, since water table depth is not the main controlling factor of aboveground biomass, then the variation of aboveground biomass may be related to light intensity, soil nutrients, and temperature, which have been proven to have a stronger constraint on aboveground biomass than water table depth in many studies [13, 37, 38, 41–43]. However, soil nutrient availability should be excluded in our study because soil fertility (including organic matter, total N and P) decreased as flooding depth decreased [26], opposite to the pattern of aboveground biomass.

### 4.4 The relationships among height, species richness and aboveground biomass

Trait-diversity relationships are particularly valuable to identify the underlying mechanisms of species diversity pattern along environmental gradient and to provide subsequent insights into the productivity-diversity relationship [8]. When communities were combined, community type and community mean height also affect species richness (Table 5). Community type mainly refers to the functional type of dominant species, e.g. graminoid, Carex in our study, and it is also one of functional traits. This further demonstrates that the method of functional traits is a powerful tool for identifying the underlying mechanisms of species richness along environmental gradients. Simultaneously, this is supported by the light competition hypothesis; plant height is a major determinant of the species’ ability to compete for light; thus, high dominant species outshade the small species surrounding it, and decrease the species richness of the community. This negative effect of community average height on species richness was also demonstrated by another study conducted by Gaudet and Keddy [44], who showed that the performance of plants grown in different neighbouring communities was negatively related to neighbour height.

Trait-function relationships are important to explain and predict the effect of biodiversity on ecosystem function and identify ecosystem process information [8]. For each community in our study, dominant species height is the only significant variable which affect aboveground biomass, and explained about 18–58% variation (Table 4, R^2^ from 0.18 to 0.58). This result is similar to the correlation found by Whitbeck and Grace [45] in Texas marshes (R^2^ = 0.35), and was a much weaker correlation than was observed by Bhattacharjee et al. [46] in the same habitat (R^2^ = 0.69). When the three communities were combined, community type and dominant species height commonly affect aboveground biomass, and this demonstrated that functional traits of dominant species are key factors which affect aboveground biomass. This is consistent with the ‘mass ratio hypothesis’ (which assumes that functional traits of the dominant species determine ecosystem function) [47] and has been demonstrated by several studies [12, 17].

The debate about the shape of the productivity-diversity relationship is still open, and many different types of relationships have been empirically found [48]. Our results showed no significant biomass-species density correlation, which is consistent with the findings of several previous wetland studies [49–51], although others have detected a humped relationship in such habitats [52, 53]. We speculate that these weak correlation between species richness and aboveground biomass may be related to the limited range of biomass values within a community, which is too narrow to demonstrate an underlying unimodal relationship.

## Conclusions

This study investigated three wetland vegetation communities along a flooding gradient in Northeast China and constitutes the first comprehensive assessment of the water regime effects on and the interrelations among plant height, species richness, and aboveground biomass. These findings indicate that if habitat drying of these herbaceous wetlands in this region continues, then two *Carex* marshes would be replaced gradually by *C*. *angustifolia* wet meadow in the near future. This will lead to a reduction in biodiversity and an increase in productivity and hence the carbon budget. Meanwhile, functional traits must be considered, and should be a focus of attention in future studies on the species diversity and ecosystem function in this region.
